# An Advance Care Planning Group Visit Intervention for Individuals With Mild Cognitive Impairment

**DOI:** 10.1093/geroni/igaa057.782

**Published:** 2020-12-16

**Authors:** Andrea Daddato, Prajakta Shanbhag, Brianne Bettcher, Hillary Lum

**Affiliations:** 1 University of Colorado School of Medicine, Parker, Colorado, United States; 2 University of Colorado School of Medicine, Aurora, Colorado, United States

## Abstract

Among older adults without cognitive impairment, a novel advance care planning group visit (ACP-GV) intervention increased ACP documentation and readiness to engage in ACP. A key question is whether an intervention can be adapted to support people with mild cognitive impairment (MCI) and a family care partner. We used a human-centered design process, rapid-cycle prototyping, and qualitative methods to adapt an ACP-GV intervention to individuals with MCI and a study partner. In 2019, we convened a longitudinal cohort of six patient-study partner stakeholders in three focus groups to suggest intervention adaptations. We also conducted a single arm study of four ACP-GV interventions (n=13 dyads total) that were iteratively refined with input from the longitudinal focus groups and intervention participant feedback. Decision tools, resources and videos were used to describe the concept of ACP and flexibility in selecting a medical decision maker. Many ACP-GV participants strongly agreed that the group discussion gave them useful information (81%) and would recommend the ACP-GV to a friend (85%). Pre- and post-ACP readiness surveys indicated that participants were significantly more ready to talk to their medical decision maker about ACP (p=0.028), while study partners perceived their loved ones less ready to speak to their doctor about ACP following the intervention (p=0.031). Use of rapid prototyping allowed testing of different resources and tools aimed at helping individuals with MCI and their study partners discuss ACP. Future work is needed to understand the feasibility of implementing an ACP-GV intervention for individuals with MCI into clinical settings.

